# Selection and Characterization of Tau Binding ᴅ-Enantiomeric Peptides with Potential for Therapy of Alzheimer Disease

**DOI:** 10.1371/journal.pone.0167432

**Published:** 2016-12-22

**Authors:** Christina Dammers, Deniz Yolcu, Laura Kukuk, Dieter Willbold, Marcus Pickhardt, Eckhard Mandelkow, Anselm H. C. Horn, Heinrich Sticht, Marwa Nidal Malhis, Nadja Will, Judith Schuster, Susanne Aileen Funke

**Affiliations:** 1 Institute of Complex Systems (ICS-6), Forschungszentrum Jülich, Jülich, Germany; 2 Institut für Physikalische Biologie, Heinrich-Heine-Universität, Düsseldorf, Germany; 3 Deutsches Zentrum für Neurodegenerative Erkrankungen (DZNE), Bonn, Germany; 4 CAESAR Research Center, Bonn, Germany; 5 Max Planck Institute for Metabolism Research, Köln, Germany; 6 Institut für Biochemie, Friedrich-Alexander-Universität Erlangen-Nürnberg, Erlangen, Germany; 7 Bioanalytik, Hochschule für angewandte Wissenschaften, Coburg, Germany; Consejo Superior de Investigaciones Cientificas, SPAIN

## Abstract

A variety of neurodegenerative disorders, including Alzheimer disease (AD), are associated with neurofibrillary tangles composed of the tau protein, as well as toxic tau oligomers. Inhibitors of pathological tau aggregation, interrupting tau self-assembly, might be useful for the development of therapeutics. Employing mirror image phage display with a large peptide library (over 10^9^ different peptides), we have identified tau fibril binding peptides consisting of d-enantiomeric amino acids. d-enantiomeric peptides are extremely protease stable and not or less immunogenic than l-peptides, and the suitability of d-peptides for *in vivo* applications have already been demonstrated. Phage display selections were performed using fibrils of the d-enantiomeric hexapeptide VQIVYK, representing residues 306 to 311 of the tau protein, as a target. VQIVYK has been demonstrated to be important for fibril formation of the full lengths protein and forms fibrils by itself. Here, we report on d-enantiomeric peptides, which bind to VQIVYK, tau isoforms like tau^3RD^ (K19) as well as to full lengths tau fibrils, and modulate the aggregation of the respective tau form. The peptides are able to penetrate cells and might be interesting for therapeutic and diagnostic applications in AD research.

## Introduction

Alzheimer disease (AD) is the most common cause for dementia and a major global cause of disability and dependency, raising significant personal and economic issues. In 2013, an estimated 44.4 million people worldwide suffered from dementia, and this number is expected to increase to 135.5 million in 2050 (http://www.alz.co.uk/research). Therapeutic options for AD are currently limited. Palliative treatment can partially reduce the symptoms, and cognitive functions can be modestly maintained temporarily [1]. Acetylcholine esterase inhibitors like Donepezil, Galantamine and the NMDA receptor antagonist Memantine have been approved for clinical use in the treatment of cognitive symptoms, but no disease altering substances are currently available [2–4].

Classical hallmarks of AD are aggregated protein deposits, i.e. senile plaques, composed of the Amyloid-β (Aβ) peptide, and neurofibrillary tangles, composed of tau protein in the brain tissue, as already described by Alois Alzheimer in the year 1907 [5].

Tau is a highly soluble microtubule associated protein playing a central role in stabilization and organization of microtubules. It is abundant in neurons of the central nervous system, where it can be particularly found in axons. There are six isoforms of tau in the human cerebrospinal fluid, resulting from alternative splicing of 16 exons of the microtubule associated protein tau (MAPT) gene, located on chromosome 17q21. The isoforms can be divided into two major groups: 1) 4R tau, that contains 4 microtubule binding repeats (each 31–32 amino acids long), and 2) 3R tau, that contains 3 microtubule binding repeats (lacking repeat 2; R2) [6].

Tau protein aggregates pathologically in AD, but also in other neurodegenerative diseases [7,8]. The distribution pattern of tau aggregates in the brain correlates well with cognitive decline in AD and can be used for staging of the disease [9]. It is currently hypothesized that amyloids propagate from cell to cell in a prion like manner [10,11].

Assembly of tau protein into paired helical filaments (PHFs) depends on a short sequence motif, 306-VQIVYK-311, also termed PHF6, which is located at the beginning of the third internal repeat. This motif coincides with the highest predicted β-structure potential in tau. Point mutations in the hexapeptide region can decrease or increase aggregation, depending on the change in β-propensity [12,13].

A variety of potential therapeutic substances for AD, like Aβ production inhibitors, Aβ aggregation inhibitors or Aβ antibodies were tested successfully *in vitro* or in AD animal models, but many have failed in clinical trials due to side effects, or they have failed to demonstrate significant therapeutic success [1]. The relationship between AD, Aβ and tau pathology is poorly understood, but recent results suggest that tau is not simply a downstream process of Aβ aggregation [14], and substances that inhibit tau aggregate formation might be interesting for AD therapy development.

A variety of tau aggregation inhibiting substances have been described [15], but only one compound, a derivative of methylene blue (LMTX), is at present under clinical investigation (Phase III, www.alzorg.com). Currently, only two peptide compounds addressing tau pathology are known. Davunetide (DAP) is an eight amino acid peptide derived from the activity-dependent neuroprotective protein ADNP. It decreases tau phosphorylation and Aβ levels in tau transgenic mice and 3 x transgenic (tg)-AD mice [16,17]. The intranasal formulation AL-108 was tested to be safe in a 12 week phase placebo controlled trial in mild cognitive impairment (MCI) patients [18]. However, DAP does not act as a tau aggregation inhibitor. In 2011, Sievers et al. developed a d-amino acid inhibitor of tau aggregation. The peptide TLKIVW was designed on the tau 306-VQIVYK-311 steric zipper template in order to block the addition of additional layers of VQIVYK. The apparent dissociation constant between the d-peptide and VQIVYK was estimated to be 2 μM, and the inhibitor prevented aggregation of PHF6 as well as of the tau constructs K12 and K19, both lacking the second repeat R2 [19].

Here, we describe the selection and characterization of novel tau fibril binding d-enantiomeric peptides by mirror image page display. d-peptides were already shown to be suitable for *in vivo* application as they are protease resistant and less immunogenic than the respective l-peptides [20–22]. In addition, it was demonstrated that therapeutic peptides can be absorbed systematically after oral administration [23]. Earlier, we reported on the identification and characterization of Aβ42 binding d-enantiomeric 12-mer peptides, also generated by mirror image phage display [24–26]. The d-peptide D3 (amino acid sequence rprtrlhthrnr) modulated Aβ aggregation and reduced Aβ42 cytotoxicity in cell culture. In APP/PS1 mouse models, D3 reduced plaque load and cerebral inflammation after oral treatment, and the cognitive behavior was significantly increased if compared to untreated control mice [27]. The tau binding d-peptide compounds described here might be an interesting alternative to Aβ binding substances, or might be combined to achieve a drug with dual mode action.

## Materials and Methods

### Peptides

All peptides were obtained commercially as reversed phase high performance liquid chromatography purified products (JPT Biotech, Berlin, Germany). For attachment of the FAM-label, an additional lysine was added C-terminally to the respective peptide. The d- or l-enantiomeric PHF6 peptide was obtained N-terminally acetylated, as charge distribution has been shown to have a profound effect on the propensity of peptides to aggregate into filaments and in the absence of N- and C-terminal charges, the blocked peptides closely mimic peptide segments within the protein structure. Unacetylated PHF6 does not aggregate *in vitro*[28–30].

### PHF6 fibrillization for phage display selection

The lyophilized d- or l-enantiomeric acetylated PHF6 peptide was solved in hexafluoro-2-propanol (HFIP) to a molarity of 1.5 mM, respectively, to be used as peptide stock solution. PHF6 fibrillization was started after dilution of 50 μM PHF6 peptide in 50 mM NaPi, pH 7.0. PHF6 fibrillization was monitored using the ThioflavinT (ThT) assay. For ThT analysis, 50 μL of the sample were separated and 5 μL ThT (10 μM) were added. The fluorescence measurement was performed in black 96-well polystyrene microtiter plates (Thermo-Fischer Scientific, Waltham, USA) at the fluorescence photometer POLARstar optima (BMG-Labtechnologies, Ortenberg, Germany), excitation/emission wavelengths were set at 440/490 nm.

### Phage display selection

The fibrillized d- or l-enantiomeric PHF6 peptide, prepared as described above, was immobilized on polystyrene 96-well microtiter plates (Thermo-Fischer Scientific, Waltham, USA). The assay plate to be coated was shaken for 1 hour at room temperature with gentle agitation. Until the selection started, the coated assay plate was stored at 4°C. Phage display was performed using the Ph.D.-12 Phage Display Peptide Library Kit (New England Biolabs, Ypswich, USA) according to the instructions of the manufacturer.

### Single clone ELISA

The single-clone ELISA was required to identify single phages showing the strongest binding to PHF6 fibrils. To perform ELISA analysis with phage supernatants, polystyrene 96-well microtiter plates (Thermo-Fischer Scientific, Waltham, USA) were coated with PHF6 fibrils, prepared as described above, but in a concentration of 100 μg/mL (126.6 μM). As controls, only 50 mM NaPi, pH 7.0, without PHF6 fibrils was applied. 2 x 100 μL of each amplified phage suspension was mixed with 100 μL blocking buffer (10 mg/mL BSA in TBS) and incubated with gentle agitation for 20 minutes at room temperature to eliminate possible BSA-binding phages. Then, the samples were transferred to PHF6 fibril coated wells and to the control wells. Subsequently, the plates were incubated for 1 hour at room temperature with gentle agitation, followed by a 5-fold washing step with TBST (0.1% Tween-20 in Tris-buffered saline (TBS, 50 mM Tris, 150 mM NaCl, pH 7.6). The HRP/Anti-13 Monoclonal Conjugate kit stock solution (GE-Healthcare, Little Chalfont, UK) was diluted 1:5000 and the antibodies were incubated on the plates for 1 hour at room temperature, followed by 6-fold washing with TBST. A 3,3',5,5'-tetramethylbenzidine (TMB) tablet (Sigma-Aldrich, St. Louis, Missouri, USA) was dissolved in 1 mL dimethyl sulfoxide (DMSO) and mixed with 9 mL phosphate-citrate buffer (Sigma-Aldrich, St. Louis, Missouri, USA). 100 μL of the solution were transferred to the according sample wells. The horseradish peroxidase reaction was stopped by addition of 100 μL 2M H_2_SO_4_. The photometric quantification was carried out at OD_450nm_ using a POLARstar optima microtiter plate reader (BMG-Labtechnologies, Ortenberg, Germany).

### Full lengths tau protein expression and purification

The gene for human tau isoform 6, *tau40*, has 1323 base pairs, encodes a protein of 441 amino acids, which contains 4 terminal repeats which form the core of the microtubule binding domain [31]. The respective tau40 gene was commercially synthesized and cloned into the pET28A(+) vector (Genentech, San Francisco, USA) via *Nco*I / *Xho*I restrictions sites. The C-terminal His-tag was deleted by addition of a stop codon before the encoding sequence. Protein expression and purification were performed according to Margittai et al. [32].

### Fluorescence assays for inhibition of fibrillization

For PHF6 fibrillization inhibition assays, 25 μM PHF6 out of a 1.5 mM HFIP stock was incubated in PBS buffer. After 2 days of incubation, ThT in a concentration of 10 μM was added to the pre-incubated samples, followed by 30 minutes final incubation at room temperature. A 384-well microtiter plate was used for analysis; the wells were treated with gaseous nitrogen and 50 μL sample volume were pipetted into each well. To quantify β-sheet based fibril formation, the relative fluorescence intensity was read out at 440/490 nm at a POLARstar optima microtiter plate reader (BMG-Labtechnologies, Ortenberg, Germany) at room temperature. The relative fluorescence was normalized to the positive controls, which were set to be 100%, respectively.

Because of its high hydrophilicity, full lengths tau protein does not fibrillize spontaneously *in vitro*, but in presence of acidic lipid micelles, it forms β-sheet based fibrils [33]. To obtain fibrils, purified recombinant tau protein in 5 μM concentration, prepared as described above, was incubated with 100 μM arachidonic acid (AA) in PBS buffer and incubated at room temperature for a minimum of 24 hours. Negative controls were tested by incubation of the recombinant protein in the absence of AA. To characterize the potential of the d-peptides to inhibit tau fibril formation, they were added to the aggregation mix described above in a molar ratio of 1:1 and 1:10 (tau:peptide), respectively.

The dose-dependent inhibition of tau aggregation was performed in the Mandelkow lab using Thioflavin S (ThS) and the tau^3RD^ construct K19, as already described before [34,35].

### Dynamic light scattering

Dynamic light scattering (DLS) enables the determination of the hydrodynamic radius of molecules and is frequently used to investigate the influence of fibrillization inhibiting substances. Recombinant tau protein was dissolved in PBS and in presence of 100 μM AA to a concentration of 1 mg/ml (21.8 μM). The respective peptide was added in a molar ratio of 1:10 (tau to peptide). As a positive control, tau without peptides was tested after addition of AA, and as negative control, it was tested without addition of neither peptides nor AA. The state of aggregation was determined after 24 hours. DLS measurements were carried out with a DynaPro DLS system (Protein Solutions, Lakewood, NJ, USA) at 20°C, a fixed angle (90°) and a cuvette path lengths of 3 mm. The acquisition time was 5 seconds with a read interval of 1 second and laser power 100% at 20°C, using a 655.6 nm (13 mW) laser. Analysis and averaging of the collected data was performed with the software Dynamic V6 (Protein Solutions). By using calculated autocorrelation functions, a regularization fit was performed in order to obtain the size distribution profile. The histograms of all measurements showed mostly monodisperse size distributions. The calculated hydrodynamic radii of the negative control, the tau monomer without addition of peptides nor AA, was used as the standard. The radii of tau in the presence of AA and peptides were normalized to the negative control and plotted as a quotient in logarithmic scale. One should keep in mind that the hydrodynamic radii are approximated on the basis of spheres. Mature fibrils in fact often display a rod-like or fibrous morphology, and it should be noted that in addition to this approximation the ability to accurately extract particle size with dynamic light scattering diminishes with increased aggregate size and conformational heterogeneity [36].

### Penetration of peptides into cells

The peptides were obtained with a 6-Carboxyfluorescein (FAM) label, linked via a C-terminal lysine, to assay their ability to enter N2a cells expressing a variant of tau protein (construct tau^4RDΔK280^, derived from the 4-repeat domain) in a regulatable fashion [37]. The cells were incubated in the presence of the peptides for two hours (short term uptake) and four days (long term uptake and stability) at concentrations of 60 μM. After incubation the cells were fixed on glass cover slips and monitored by a confocal microscope (Fluoview 1000, Olympus). The localizations of the d-peptides were detected by their FAM-label (excitation: 488 nm, emission: 520 nm). In addition, cell nuclei were stained with TOPRO3 dye (excitation: 633 nm, emission: 660–670 nm). Representative pictures were taken with magnifications of 10-times for overview and 60- and 120-times for more detailed analysis.

### *In silico* modelling

Modeling of the APT and TD28 peptides complexes with PHF6 was guided by a previous model of PHF6 with the d-peptide TLKIVW published by Sievers et al. [19]. We reconstructed the complex based on the crystal structure of unliganded PHF6 (PDB code 2ON9 [38]) by docking the d-peptide TLKIVW in an extended conformation to PHF6 using the same hydrogen bond register described by Sievers et al. [19]. APT and TD28 were then modeled in the same extended geometry present in the TLKIVW peptide. The binding register was set according to the position of a conserved basic residue present in all three peptides. Modeling was performed with Sybyl 7.3 (Tripos Inc., St. Louis, USA) and UCSF Chimera [39]. Visual inspection of the complexes between the PHF6 oligomers and the docked peptides was performed with VMD [40].

## Results

### Selection of PHF6 binding peptides

**Fig 1:**
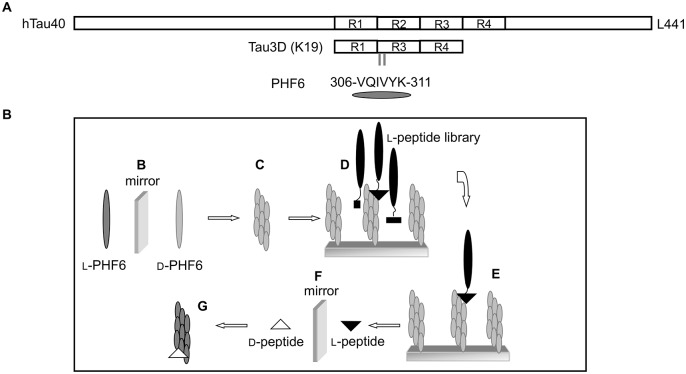
Tau constructs and principle of the mirror image phage display selection. (A) The largest human tau isoform in the central nervous systems contains four microtubule binding repeats, whereas the tau construct tau^3RD^ (K19) lacks repeat 2 [42]. PHF6, consisting of amino acids 306 to 311, is located at the beginning of repeat three. (B) The d-enantiomeric form of PHF6 was synthesized. (C) After fibrillization, the d-enantiomeric fibrils were used for phage display (D). (E) An l-peptide, binding to the d-enantiomeric PHF6 fibrils, was selected. (F) The d-enantiomeric version of the selected l-peptide was synthesized, which (G) will bind to the l-enantiomeric form of the target, regular PHF6 fibrils.

As it was hardly possible to synthesize d-enantiomeric full lengths tau protein (lengths 441 amino acids) for mirror image phage display, we realized the selections of a peptide library encoding more than 1 x 10^9^ different random 12-amino acid sequences with fibrils of the d-enantiomeric hexapeptide VQIVYK (d-PHF6) as the target. In addition, we performed selections with l-enantiomeric PHF6 fibrils under same conditions. The fragment VQIVYK has been demonstrated to be indispensable for tau aggregation and forms itself fibrils, [12,13], therefore we expected the resulting peptides to bind fibrils of the full lengths tau protein. The location of the PHF6 sequence in the tau protein is depicted in Fig 1A. The principle of the mirror image phage display selection is shown in Fig 1B. To prepare PHF6 fibrils for the selection procedure, PHF6 was HFIP treated and incubated in sodium phosphate buffer for at least two hours. Fibrillization was monitored using ThT-fluorescence assays, and the resulting fibrils were immobilized in microtiter plates. Thioflavin is a benzothiazole dye that exhibits enhanced fluorescence upon binding to amyloid fibrils. The quantity of the bound dye is determined by measuring the relative fluorescence [41].

After four rounds of biopanning, single phages of the finally enriched phage dilution were tested for their ability to bind PHF6 fibrils using single phage ELISA, and the peptide sequences of promising phages were determined by DNA sequence analysis of the respective genome region. Interestingly, not one single dominating amino acid sequence or consensus sequence was found in 23 sequences, but some sequences were selected twice or three-fold. 10 peptides were chosen for further characterization and for testing their potential to inhibit tau aggregation. The peptide sequences are listed in Table 1. All peptide sequences found after selection were compared to already known peptide sequences listed in the SAROTUP database (“Scanner And Reporter Of Target-Unrelated Peptides", a suite of web tools which can be used to scan, report and exclude possible target-unrelated peptides from biopanning results: http://immunet.cn/sarotup/) and the PepBank database (database of peptides based on sequence text mining and public peptide data sources: http://pepbank.mgh.harvard.edu/), but no hits were documented.

**Table 1:** Selected peptides tested for their potential to inhibit PHF6 fibrillization using ThT assay.

Name	Sequence	Selection	Inhibition of PHF6 fibrillization
**APT**	d-aptllrlhslga	d-vqivyk	+
**KNT**	d-kntpqhrklrls	d-vqivyk	+
**TL28**	l-TTSLQMRLYYPP	l-vqivyk	+
**TD28**	d-ttslqmrlyypp	d-vqivyk	+
**TD28rev**	d-ppyylrmqlstt	d-vqivyk	+
**Positive Control**	d-tlkivw [19]	d-vqivyk	+
**TD2**	d-stfdpltlhktr	d-vqivyk	-
**TD12**	d-dknhvspvqiyk	d-vqivyk	+/-
**TL8**	l-sshnvnggtwgs	l-vqivyk	+/-
**TL18**	l-mnlsngevynak	l-vqivyk	-

All peptides listed in the table were tested for their potential to inhibit PHF6 fibrillization using ThT assay. (+/-) indicates comparably low inhibition of PHF6 fibril formation, (-) indicates that the peptide showed no effect on fibril formation.

### *In vitro* characterization of PHF6 and tau aggregation inhibition

**Fig 2:**
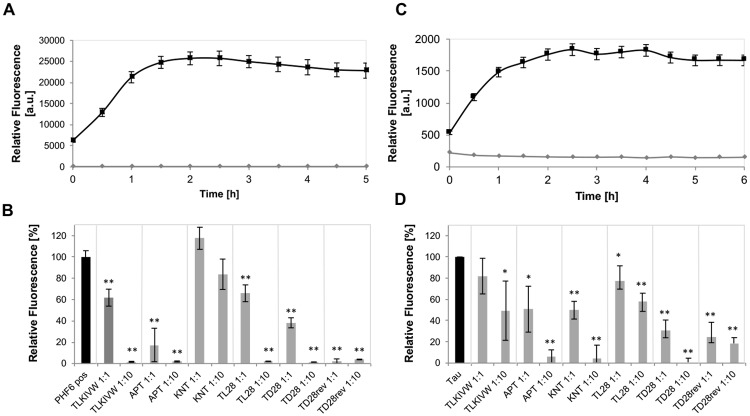
ThT assays for characterization of the peptides abilities to inhibit fibril formation. (A) PHF6 fibrillizes spontaneously within 2 hours. The assay was performed using 25 μM PHF6 peptide in PBS with 10 μM ThT (black line). PBS and 10 μM ThT without addition of PHF6 was used as control (grey line). Fluorescence was measured at 490 nm in relative units (mean +/- standard deviations of results, five replicates per run. (B) PHF6 fibrillization was inhibited by the selected peptides. The respective peptide was added in concentrations of 25 or 250 μM to 25 μM PHF6 samples, respectively. Upon addition of ThT, fluorescence was measured at 490 nm in relative units (mean +/- standard deviations of results, five replicates per run). The relative fluorescence for the PHF6 only control after 2 hours incubation (saturation level) was set at 100%. *: p < 0.05; **: p < 0.0005; students t-test. (C) Recombinant full lengths tau protein fibrillizes under assay conditions. 5 μM tau, 100 μM AA and 10 μM ThT were incubated in PBS (black line). As a negative control, 5 μM tau was incubated in PBS with ThT, without addition of AA. Fluorescence was measured at 490 nm in relative units (mean +/- standard deviations of results, five replicates per run). The relative fluorescence of a PBS sample with 100 mM AA was subtracted. (D) ThT assays were employed to characterize the abilities of peptides to inhibit full lengths tau fibril formation. The respective peptide was added in concentrations of 50 μM peptide to 5 μM tau and 100 M AA. Upon addition of ThT, fluorescence was measured at 490 nm in relative units (mean +/- standard deviations of results, five replicates per run). The relative fluorescence for the tau and AA control without peptides and after 2.5 hours incubation (saturation level) was set at 100%. *: p < 0.05; **: p < 0.0005; students t-test.

To investigate the potential of the selected peptides to inhibit fibrillization of PHF6, the tau construct K19 and full lengths tau, we performed Thioflavin fluorescence assays. The fibril formation of acetylated PHF6 was monitored in the presence and absence of a one- or tenfold molar access of the respective peptide. The fluorescence of the control sample without peptide reached a saturation level after two hours of incubation (Fig 2A), therefore, the value after 2 hours was set to be 100%. The results of the peptides selected in this study were compared to the performance of the TLKIVW peptide described by Sievers et al. [19]. As listed in Table 1, the peptides TD2, TD12, TL8, and TL18 did not show any significant effect on PHF6 fibril formation. TL20 even seemed to increase fibrillization of PHF6. These respective peptides were not used for further investigation. As can be seen in Fig 2B, all other selected peptides reduced PHF6 fibril formation significantly, especially in a ratio of 1:10. The peptide KNT did not inhibit PHF6 fibrillization in a ratio of 1:1 and had only a minor effect in a ratio of 1:10. None of the peptides showed self-fibrillization tendencies in the control sample without PHF6 under assay procedure conditions. To investigate if the relatively small, 12-meric peptides could also inhibit full lengths tau aggregation, similar Thioflavin assays as described above were performed with full lengths tau. Tau aggregation was initiated by addition of arachidonic acid (AA) (see Fig 2C), and tau in a concentration of 5 μM was incubated in absence or presence of the respective peptide in a molar ratio of 1:1 or 1:10. After three hours of incubation, the fluorescence of the tau-only control sample reached a saturation level, and the value of fluorescence was set to 100%. In this assay, we could demonstrate a weak effect of the TLKIVW peptide, all peptides selected in this study clearly inhibited fibril formation of the tau protein. As the d-peptides were selected using PHF6 fibrils, we ordered FAM labeled versions of the promising peptides to confirm their binding to full lengths tau protein and its fibrils. For APT-Lys(FAM)-NH_2_, KNT-Lys(FAM)-NH_2_, LPS-Lys(FAM)-NH_2_, TD28-Lys(FAM)-NH_2_, TD28rev-Lys(FAM)-NH_2_ as well as for d-tlkivw-Lys(FAM)-NH_2_ binding could be demonstrated for tau monomers as well as for tau fibrils. No significant difference could be detected between the binding to the different conformers. Except for APT-Lys(FAM)-NH_2_, all peptides showed a minimal preference for tau monomers, probably due to better accessibility of the PHF6 motif, which might partly be hidden inside the fibrils. The ELISA signal was highest for KNT-Lys(FAM)-NH_2_, TD28-Lys(FAM)-NH_2_, as well as the positive control peptide d-tlkivw-Lys(FAM)-NH_2_, see S1 Fig.

**Fig 3:**
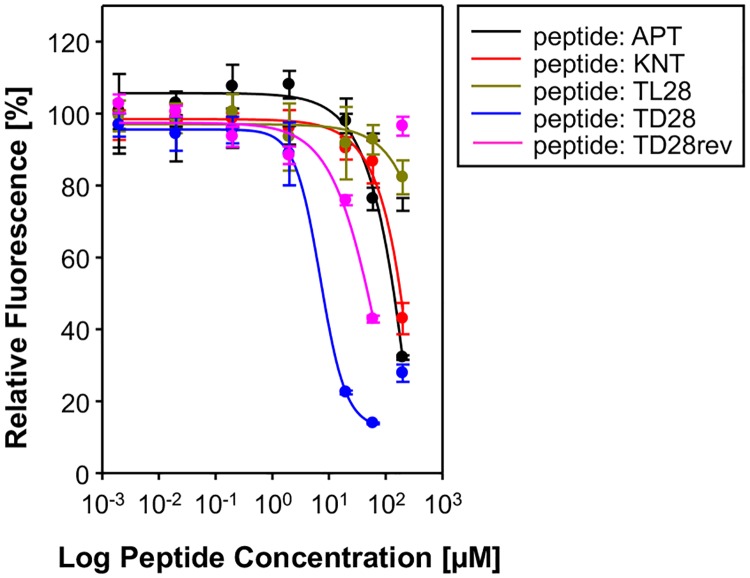
ThS inhibition assays were performed *in vitro* using the tau^3RD^ construct K19. Dose-response curves for inhibition of tau aggregation were plotted as percentage of the untreated control. Samples treated with d-peptides show a decreasing ThS signal at increasing concentrations, indicating an inhibition of tau aggregation.

Next, we tested the peptides APT, KNT, LPS, TL28, TD28, and TD28rev for their ability to inhibit the aggregation of tau^3RD^ (three repeat domain construct of tau protein, also termed K19, see Fig 1) *in vitro* as measured by ThS fluorescence [35,42]. In addition, we have determined the half maximal effect concentrations of the peptides. K19 was chosen because it fibrillizes reliably at low micromolar concentrations in the presence of the polyanionic cofactor heparin and resembles Alzheimer paired helical filaments [12,13]. The results can be seen in Table 2 and Fig 3. The half maximal effects were achieved at the following concentrations: TD28 (7.9 μM), TD28rev (96.2 μM), APT (139.6 μM), KNT (182.8 μM), LPS (> 200 μM), TL28 (> 200 μM).

**Table 2:** Half maximal effect concentrations.

Peptide name	Half maximal effect concentration [μM]
**TD28**	7.9
**TD28rev**	96.2
**APT**	139.6
**KNT**	182.8
**TL28**	>200

Inhibition curves of tau^3RD^ fibril formation were performed *in vitro* by ThS assays. Tau^3RD^ (K19) samples treated with the d-peptides showed a decreasing ThS signal at increasing peptide concentrations, indicating an inhibition of aggregation.

At peptide concentrations > 100 μM, an increasing ThS fluorescence for the cases of TD28 and TD28rev were observed. This might be explained by direct binding of ThS to these peptides (possibly due to self-aggregation). In contrast to the PHF6 aggregation inhibition assay, this effect was already visible for TD28rev and ThS in the absence of tau protein under this assay procedure conditions (data not shown).

**Fig 4:**
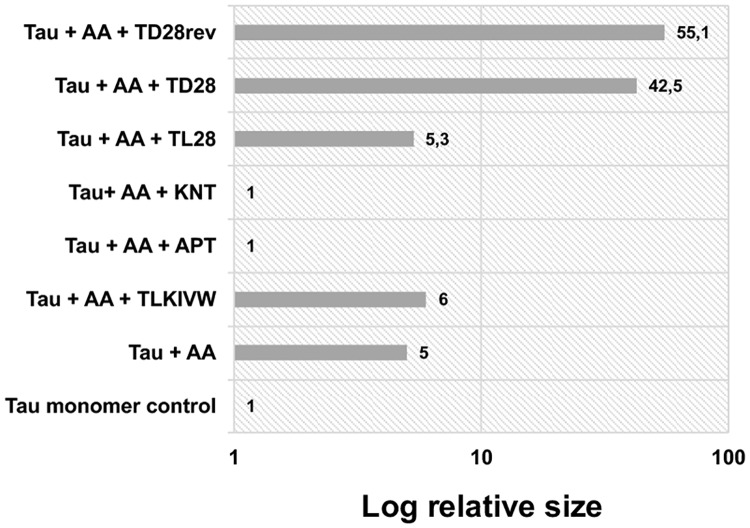
Effect of the selected peptides on tau aggregation, measured by DLS. Recombinant tau was dissolved in PBS to a concentration of 21.8 μM (1 mg/ml). As positive control, AA was added to a concentration of 100 μM. The peptides were added in a ratio of 1:10 (tau:peptide) and 100 μM AA. All samples were incubated for 24 hours. DLS was performed with an acquisition time of 5 seconds for 3 minutes at room temperature. The ratio of the hydrodynamic radii of tau protein in the presence of the peptides to tau fibrils (positive control) was calculated. The error was below 10% for all measurements, the mass was ≥ 99%.

We wanted to confirm the results of the ThS studies by DLS (see Fig 4). Full lengths tau protein was dissolved to 1 mg/mL in PBS (21.8 μM). 100 μM AA were added as well as the respective peptide in a molar ratio of 1:10 (tau:peptide). The state of aggregation was determined at different time points from 1 hour to 24 hours. While peptides APT, KNT and LPS inhibited formation of tau aggregates, TD28 and TD28rev induced the formation of large particles, again possibly due to self-aggregation under the given experimental conditions. TL28 and the TLKIVW peptide did not show any inhibitory effects.

### Ability of the peptides to penetrate cells

**Fig 5:**
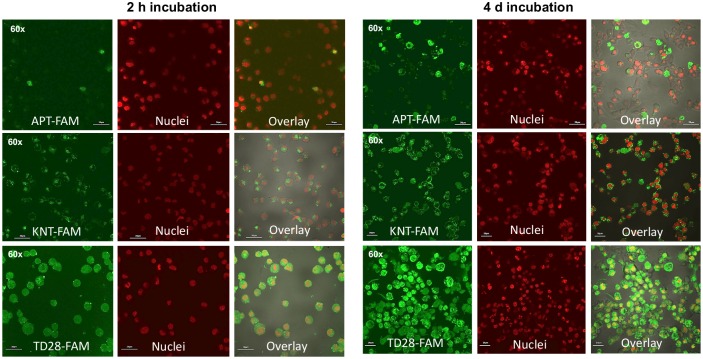
Inducible N2aTau^4RDΔK280^ cells were incubated with 60 μM of the respective, FAM-labeled peptide for two hours and four days. After incubation, the cells were fixed on glass cover slips and the locations of the respective peptide were detected by the FAM-label (green). In addition, cell nuclei were stained with TOPRO3 dye (red). Here, representative pictures for APT-FAM, KNT-FAM and TD28-FAM are shown in 60 times magnification. Scale bar: 30 μm.

To be effective as therapeutics, peptides will have to be able to cross the blood brain barrier and the cell membranes of neurons. In this study, we tested FAM-labeled versions of the d-peptides for their ability to enter N2a cells expressing a variant of tau protein (derived from the repeat domain) in a regulatable fashion [37]. The cells were incubated in the presence of the peptides for two hours (short term uptake) and four days (long term uptake and stability) at concentrations of 60 μM. After incubation the cells were fixed on glass cover slips and monitored by a confocal microscope. The localizations of the d-peptides were detected by their FAM-label, in addition cell nuclei were stained with TOPRO3 dye. Representative pictures for APT-FAM, KNT-FAM and TD28-FAM in 60-times magnification are summarized in Fig 5. Cell compartments, where labeled d-peptides could be detected, are summarized in Table 3. Representative confocal z-stacks for peptides KNT-FAM and TD28-FAM with magnified frame 10 are shown in S2 and S3 Figs. The peptide KNT-FAM shows accumulations of punctate foci in the cytosol and separately some foci beneath the cell membrane (green dots) but no localization in the nucleus (S2B Fig, arrows). In contrast, the distribution of TD28-FAM is more diffuse over the whole cell body. The fluorescence appears near the membrane, in the cytosol, and in the nucleus (here merged with the red nucleus staining as yellow; S3B Fig, arrows).

**Table 3:** Cell penetration of the peptides.

Peptide name	2 hours incubation			4 days incubation		
	membrane	cytosol	nuclei	membrane	cytosol	nuclei
**APT**-Lys(FAM)-NH_2_	+	+/-	-	+	+	+/-
**KNT**-Lys(FAM)-NH_2_	+	+	-	+	+	-
**LPS**-Lys(FAM)-NH_2_	+	+	-	+	+	-
**TD28**-Lys(FAM)-NH_2_	+	+	+/-	+	+	+
**TD28rev**-Lys(FAM)-NH_2_	+	+	+	+	+	+

60 μM of the respective FAM labeled peptide was incubated with inducible N2aTau^4RDΔK280^ cells [37]. After incubation of 2 hours or 4 days the cells were fixed on glass cover slips and monitored by a confocal microscope. The localizations of the peptides were detected by their FAM-label. In addition, cell nuclei were stained with TOPRO3 dye. Cell compartments where fluorescence of the label was detected are summarized here. Representative example pictures with 60 times magnification are illustrated in Fig 5.

The treatment of tau expressing N2a cells with FAM-labeled d-peptides led in all cases to an accumulation of peptides in the cytoplasm, visible at the latest after 4 days of incubation. We conclude that all d-peptides are able to penetrate the cell membrane, in which the APT-FAM peptide seemed to have the worst cell penetration properties. In some cases (APT-FAM, TD28-FAM, TD28rev-FAM), there was an overlap of peptide and nuclei staining, suggesting penetration into the nucleus. On the other hand, many of the ‘peptide-positive’ cells appeared to have lost their nuclei, which may reflect potential cytotoxicity of this peptide. In addition, for TD28 and TD28rev precipitation of the peptides in the medium was observed.

To obtain first hints on the effect of treatment on the inducible N2aTau^4RDΔK280^ cells with d-peptides, we incubated the cells with 10 to 60 μM of the respective peptide in presence of THS and doxycyclin, except for the untreated control, and quantified the ThS fluorescence by fluorescence-activated cell sorting (FACS). Cells with ThS-positive aggregates were measured by the fluorescent signal intensity in the green channel (excitation: 495 nm, emission: 519 nm). The amount of ThS-positive cells in the compound-untreated DMSO control was set to 100%. The bar diagram in S4 Fig shows the relative amount of Thioflavin S positive cells when treated with increasing amounts of the tested d-peptides for 4 days, compared to the untreated control, as quantified by FACS. In the case of peptides APT and KNT there was a small decrease in the number of ThS-positive cells (e.g. 8.5% at 60 μM APT, and 9.5% at 20 μM KNT-peptide), whereas treatment with TL28 shows no reduction of ThS-positive cells. In contrast the treatment with TD28rev (10–30 μM) increased the number of ThS-positive cells up to 240% compared with the untreated control. This seems to be a non-specific effect because the whole cell population (ThS-positive and -negative cells) shifted to higher fluorescence intensities when treated with 30 μM TD28rev comparing to the control samples (data not shown).

### *In silico* analysis of binding mode

**Fig 6:**
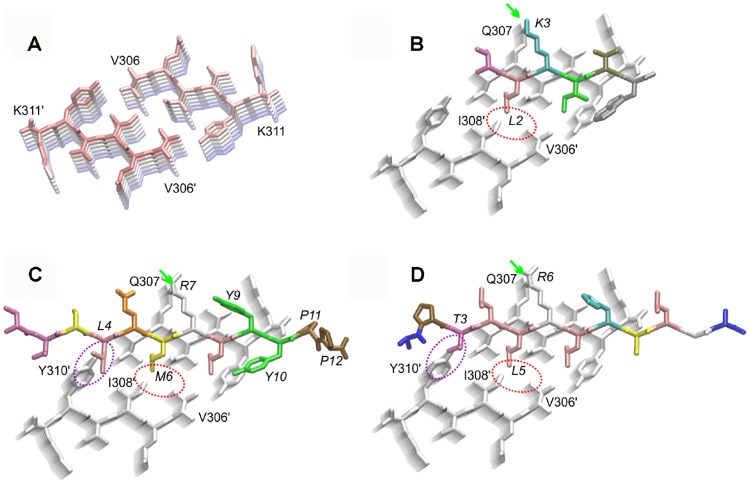
Structure of the tau fibril and binding mode of different d-peptides. (A) Crystal structure of the steric zipper formed by the PHF6 peptide. The first and last residue for each of the two stacks of interacting β-sheet is labeled. The individual layers of β-sheet within each stack are gradually colored from red to blue. (B) Binding mode of the TLKIVW peptide according to Sievers et al. [19]. The side chain interaction between K3 and Q307 is marked by a green arrow. The red ellipse denotes steric repulsion between L2 of TLKIVW and V306/I308 of the second stack of β-strands. Peptide residues are labeled in italic. (C) Binding mode of the TD28 peptide. Same type of presentation as in (B); an additional repulsive interaction formed between L4 and Y310 is highlighted by a magenta ellipse. (D) Binding mode of the APT peptide. Same type of presentation as in (B); an additional repulsive interaction formed between T3 and Y310 is highlighted by a magenta ellipse.

The crystal structure of the PHF6 peptide (residues 306–311 of tau) revealed a characteristic steric-zipper motif comprising two interacting stacks of β-strands that run perpendicular to the fibril axis [38] (Fig 6A). This structure already provided a basis for the rational design of a d-peptidic inhibitor with the sequence TLKIVW [19]. Structural models of APT and TD28 in complex with a fibrillar PHF6 oligomer show similarities to the TLKIVW-PHF6 complex (see Fig 6): For all three peptides, binding in a parallel β-sheet conformation allows the formation of stabilizing backbone hydrogen bonds to PHF6. Similar to TLKIVW, APT and TD28 can establish a favorable side chain contact between the central positively charged residue (R in APT and TD28, K in TLKIVW) and Q307 of PHF6. Sievers et al. suggested steric repulsion between L2 of TLK and V306/I308 of the second stack of β-strands as key feature blocking fibril growth [19]. The same type of clash is also formed by residues L5 of APT and M6 of TD28, which are located at the structurally equivalent position to L2 of TLKIVW (Fig 6B–6D). Compared to TLKIVW, APT and TD28 exhibit additional steric clashes with Y310 formed by L4 of TD28 and by T3 of APT (Fig 6C and 6D), suggesting that these peptides can more potently hinder PHF6 aggregation.

## Discussion

Currently, no disease modifying therapeutics for tauopathies are available. In the present study, we have demonstrated that PHF6 binding d-enantiomeric peptides, selected by mirror image phage display, inhibit PHF6 as well as full lengths tau aggregation. In addition, the abilities of the selected peptides to penetrate cells were shown.

Currently, only one peptide compound addressing tau pathology by a signaling mechanism, Davunetide (DAP), decreasing tau phosphorylation and Aβ levels in tau transgenic mice, is known [16,17]. Another d-enantiomeric peptide, TLKIVW, was rationally designed by Sievers and colleagues to inhibit tau fibril formation [19]. However, during the past years, several peptide inhibitors of Aβ aggregation have been investigated for their applicability as new therapeutic lead compounds. Only very few were proven to be effective in mouse models [43]. Despite the advantages of peptides, like high diversity, combined with their simplicity, specificity, high biological activity and low toxicity [44,45], peptides are susceptible to proteolytic degradation and in general are not stable in blood. Additionally, peptides are known not to cross membranes very well [46,47]. The direct intracellular delivery of peptides has been difficult to achieve, due primarily to the bioavailability barrier of the plasma membrane, which prevents the uptake of macromolecules like peptides by limiting their passive entry [48].

Several strategies were already applied to overcome high protease susceptibility of peptides and to improve blood brain barrier permeability. One strategy to improve the peptide stability is the use of d-enantiomeric amino acids, which are considered to be rather protease resistant and often less immunogenic than the respective l-peptides [20–22]. In addition, d-peptides were described to be absorbed systematically after oral administration [23], also shown for the Aβ oligomer precipitating d-enantiomeric peptide D3 in AD mouse models [27,49]. The PHF6 binding, d-enantiomeric peptides developed in this study were demonstrated to cross the membranes of N2a cells. The mechanism of penetration is unclear yet. Peptides do in general not cross membranes very well, but several classes of cell penetrating peptides have been discovered, including naturally occurring transcription factor domain as penetratin, HIV-Tat or synthetic cationic peptides [50–52]. However, as in our experiments, in some cases an overlap of peptide and nuclei staining was visible, suggesting penetration into the nucleus, and on the other hand, many of the ‘peptide-positive’ cells appeared to have lost their nuclei which may reflect potential cytotoxicity of this peptide, cell toxicity assays will have to be performed in future. Whether the peptides also will cross the blood brain barrier of e.g. AD mouse models, will be subject of a further future study.

Phage display selections for this study were performed using fibrils of the d-enantiomeric hexapeptide VQIVYK, representing residues 306 to 311 of the tau protein, as a target. VQIVYK was shown to be crucial for tau fibrillization and is widely used as a model for tau aggregation [19,53–56]. As expected, the selected peptides also bound to full length tau protein (S1 Fig) and inhibited the fibrillization of tau^3RD^ and the full lengths tau protein in concentrations which are typical for peptide inhibitors [19,25,27]. We also could show a slight effect of peptide treatment on inducible N2aTau^4RDΔK280^ cells, here, however, further investigation is necessary and planned for future studies.

At the moment, we only can speculate about the mechanism of action. As can be seen in S1 Fig, the peptides bind similarly well to full length tau monomers as to its fibrils. For the selection of the peptides, we have used PHF6 fibers, but this does not exclude the sequence specific binding of the resulting peptides to monomers, as conformer selectivity was not intended in the selection process.

As the peptides were selected against the VQIVYK motif, they might block the addition of further building blocks as also suggested for the TLKIVW peptide [19]. This theory is supported by molecular modeling, which suggested that APT and TD28 interact with PHF6 in an analogous fashion than TLKIVW and prevent fibril formation by a similar mechanism. Their enhanced activity against full lengths tau fibrils most likely results from the larger steric repulsion caused by additional residues of the longer APT and TD28 peptides. These additional residues cause steric repulsion within PHF6 segment (Fig 6B–6D) and probably also with adjacent residues outside the PHF6 stretch. The latter tau residues could not be included in the present model due to a lack of a suitable template structure. As the small fiber forming domain VQIVYK and its vicinity is covered by a large fuzzy coat in full lengths tau fibrils [57], which could restrict access of peptides, the inhibitory effect on fibril formation might be due to binding of the peptides to pre-fibril forms of tau, keeping them in a blocked anti-aggregant state.

With respect to the formation of additional interactions, TD28 contains an interesting candidate stretch (‘YYPP’) that is rich in prolines and aromatic amino acids (Fig 6C). This stretch might act as a tau β-sheet breaker in a similar fashion as the LPFFD peptide designed against amyloid-β [58]. However, TD28 and TD28rev showed tendencies to self-aggregate under certain experimental conditions where the peptide concentrations are high (if compared to later potential *in vivo* studies). The high self-assembly tendency of proline- and tyrosine-rich tripeptides [59] suggests that the YYPP motif of TD28/TD28rev might be involved in the formation of the larger amorphous particles experimentally observed for TD28/TD28rev, but not for the other tau ligands.

We have selected d-enantiomeric peptides in order to inhibit the fibril formation of tau. Future experimental work will be needed to reveal the mechanism of action of the peptides selected in this study and to investigate their potential for future therapeutic applications. In addition, to date, it is not clear which of the tau conformers (monomer, oligomer, fibril) is the best target for therapeutic intervention. It was recently suggested that tau oligomers are the more toxic species in AD progression and these oligomers might be involved in the spreading of tau pathology from cell to cell [10,11]. In future, we plan to select peptides, which bind specifically to tau monomers and oligomers. Similar species specific peptides were earlier developed for conformers of the Aβ peptide [60,61]. Novel tau conformer specific peptides should be used to investigate in which stage of AD the inhibition of tau aggregation is reasonable and therapeutically helpful.

## Supporting Information

S1 Fig ELISA experiment demonstrating the binding of the selected peptides, as well as of a positive control, to tau monomers and fibrils in 5 μg/mL concentration. As a negative control, PBS pH 7.4 containing 1% BSA was incubated in the wells instead of tau protein solution. After incubation with 20 μg/mL of the respective peptide wit FAM-label, a horseradish peroxidase-conjugated sheep anti FITC secondary antibody was used for detection of bound peptide. The mean of three OD values (at 405 nm) is given, as well as the standard deviation.
(TIF)

S2 Fig Representative distribution of KNT-FAM in the N2a^K18ΔK280^ cell body after 4 days of incubation with 60 μM peptide. (A) Space-resolved confocal z-stack 1–20 over 9.2 μM, step size: 0.46 μM. d-peptide: FAM staining (green): exc. 488 nm, em. 520 nm; cell nuclei: TOPRO3 staining (red): exc. 633 nm, em. 660–670 nm. (B) panel 10 of z-stack shown in A; separate localizations of KNT peptides on the membrane and in the cytosol are shown in green but no peptide localization in the nuclei is visible (arrows).
(TIF)

S3 Fig Representative distribution of TD28-FAM in the N2a^K18ΔK280^ cell body after 4 days of incubation with 60 μM peptide. (A): Space-resolved confocal z-stack 1–20 over 10.2 μM, step size: 0.51 μM. d-peptide: FAM staining (green): exc. 488 nm, em. 520 nm; cell nuclei: TOPRO3 staining (red): exc. 633 nm, em. 660–670 nm. (B): panel 10 of z-stack shown in S3A). Diffuse localization of TD28 peptide on the membrane and cytosol are shown in green whereas co localization in the nucleus appears in yellow (merge of FAM- and TOPRO3 staining; S3B arrows).
(TIF)

S4 Fig Treatment of inducible N2aTau^K18ΔK280^ cells with d-peptides. The bar diagram shows the relative amount of Thioflavin S positive cells when treated with increasing amounts of the tested d-peptides for 4 days (entry #3–16), compared to the untreated control (entry #2, set to 100%, dashed line), as quantified by FACS. In the case of peptides APT and KNT a small decrease in the number of ThS-positive cells was detected (e.g. 8.5% at 60 μM Apt, entry#5 and 9.5% at 20 μM Knt-peptide, entry#7; marked with arrows), whereas treatment with TL28 shows no reduction of ThS-positive cells. In contrast the treatment with TD28rev (10–30 μM) increased the number of ThS-positive cells up to 240% compared with the untreated control (entry#16).
(TIF)

S1 Materials and Methods This file includes supporting materials and methods for S1 and S4 Figs as well as supporting literature for S4 Fig. (DOCX)
