# Supplementary figures and images for: Selection and Characterization of Tau Binding ᴅ-Enantiomeric Peptides with Potential for Therapy of Alzheimer Disease
Source: PLoS One. 2016 Dec 22;11(12):e0167432. doi: 10.1371/journal.pone.0167432 (PMC5179029; doi:10.1371/journal.pone.0167432)

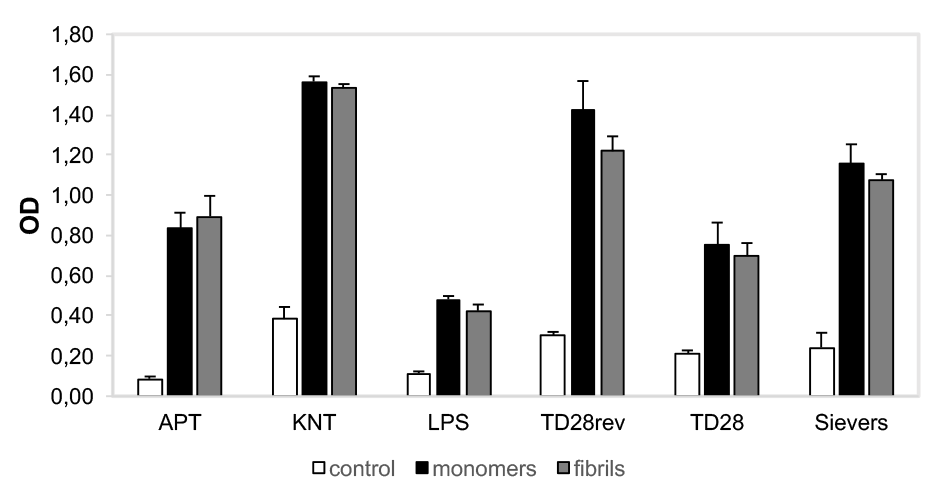

Supplement: S1 Fig — As a negative control, PBS pH 7.4 containing 1% BSA was incubated in the wells instead of tau protein solution. After incubation with 20 μg/mL of the respective peptide wit FAM-label, a horseradish peroxidase-conjugated sheep anti FITC secondary antibody was used for detection of bound peptide. The mean of three OD values (at 405 nm) is given, as well as the standard deviation. (TIF) [file pone.0167432.s001.tif]

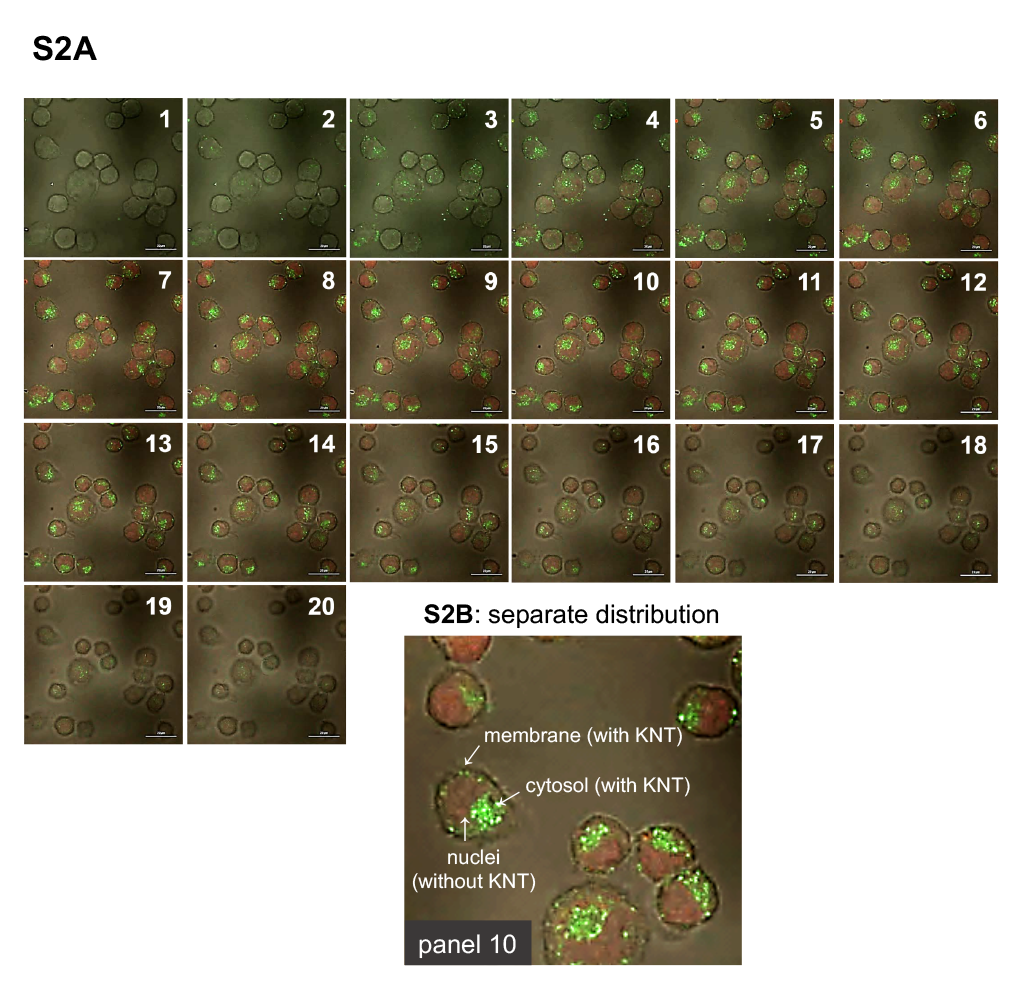

Supplement: S2 Fig — (A) Space-resolved confocal z-stack 1–20 over 9.2 μM, step size: 0.46 μM. d-peptide: FAM staining (green): exc. 488 nm, em. 520 nm; cell nuclei: TOPRO3 staining (red): exc. 633 nm, em. 660–670 nm. (B) panel 10 of z-stack shown in A; separate localizations of KNT peptides on the membrane and in the cytosol are shown in green but no peptide localization in the nuclei is visible (arrows). (TIF) [file pone.0167432.s002.tif]

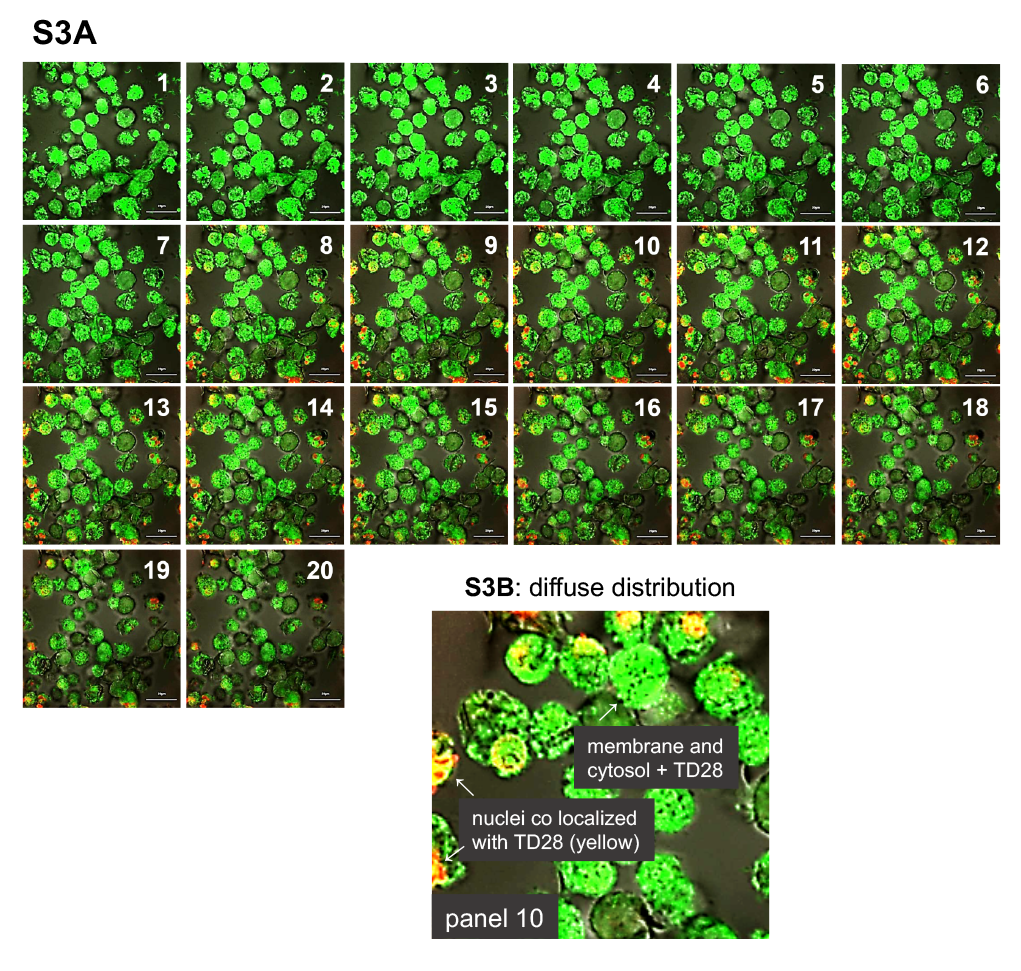

Supplement: S3 Fig — (A): Space-resolved confocal z-stack 1–20 over 10.2 μM, step size: 0.51 μM. d-peptide: FAM staining (green): exc. 488 nm, em. 520 nm; cell nuclei: TOPRO3 staining (red): exc. 633 nm, em. 660–670 nm. (B): panel 10 of z-stack shown in S3A). Diffuse localization of TD28 peptide on the membrane and cytosol are shown in green whereas co localization in the nucleus appears in yellow (merge of FAM- and TOPRO3 staining; S3B arrows). (TIF) [file pone.0167432.s003.tif]

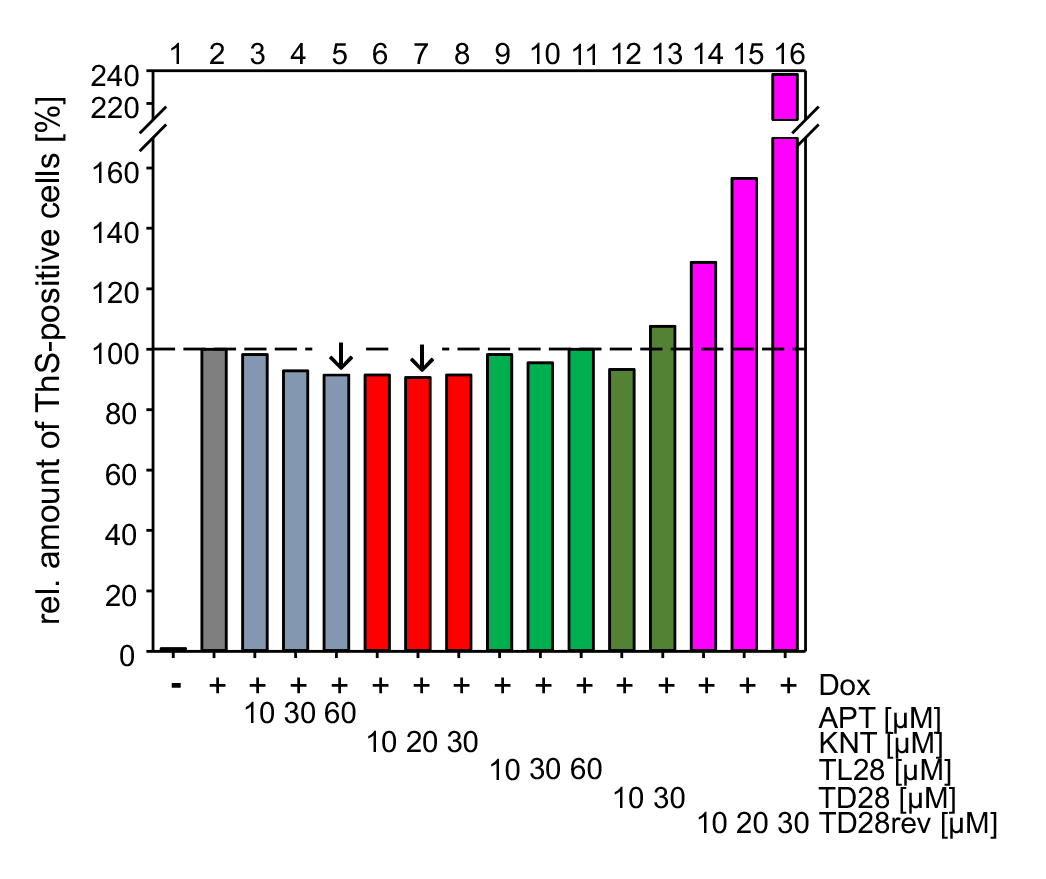

Supplement: S4 Fig — The bar diagram shows the relative amount of Thioflavin S positive cells when treated with increasing amounts of the tested d-peptides for 4 days (entry #3–16), compared to the untreated control (entry #2, set to 100%, dashed line), as quantified by FACS. In the case of peptides APT and KNT a small decrease in the number of ThS-positive cells was detected (e.g. 8.5% at 60 μM Apt, entry#5 and 9.5% at 20 μM Knt-peptide, entry#7; marked with arrows), whereas treatment with TL28 shows no reduction of ThS-positive cells. In contrast the treatment with TD28rev (10–30 μM) increased the number of ThS-positive cells up to 240% compared with the untreated control (entry#16). (TIF) [file pone.0167432.s004.tif]
