# Supplementary material for: Selection and Characterization of Tau Binding ᴅ-Enantiomeric Peptides with Potential for Therapy of Alzheimer Disease
Source: PLoS One. 2016 Dec 22;11(12):e0167432. doi: 10.1371/journal.pone.0167432 (PMC5179029; doi:10.1371/journal.pone.0167432)
Supplement: S1 Materials and Methods — (DOCX) [file pone.0167432.s005.docx]

**Supplement**

**S1 Fig Materials and Methods: Enzyme linked immunosorbent assays (ELISA)**

***Preparation of tau fibrils for ELISA***

Tau protein [tau-441 (2N4R) MW 45.9 kDa], purified as described in the main text, was used to prepare tau fibrils, the preparation of the fibrils was performed according to Barghorn et al. [1] with slight modifications: 30 µM tau protein were incubated for 48 hours with 20 µM heparin MW ≈ 5000 (Fisher scientific, Schwerte, Germany) in phosphate-buffered saline (PBS) pH 7.4 at room temperature.

***ELISA***

Polystyrene 96-well microtiter plates (Greiner Bio-One GmbH, Frickenhausen, Germany) were coated with 100 µL/well of tau monomers or fibrils in concentration of 5 µg/mL respectively, followed by incubation for 1 hour at room temperature. As control, PBS pH 7.4 containing 1 % BSA (IgG free) (Roth, Karlsruhe, Germany) was used instead of tau protein solution. After washing three times with PBS-T (phosphate-buffered saline (pH 7.4) with (0.1 %) Tween 20), and blocking for 1 h at room temperature with 1 % BSA (IgG free) (Roth, Karlsruhe, Germany) in PBS, the plate was washed three times with PBS-T. Then the peptides APT-Lys(FAM)-NH2, KNT-Lys(FAM)-NH2, LPS-Lys(FAM)-NH2, TD28-Lys(FAM)-NH2, TD28rev-Lys(FAM)-NH2 as well as d-TLKIVW-Lys(FAM)-NH2 (JPT Peptide Technologies, Berlin, Germany) were dissolved in PBS-T and added at 100 µL/well in concentration of 20 µg/mL, respectively, the plate was allowed to react for 1 h at room temperature in the dark. Subsequently, the plate was washed 3 times with PBS-T, horseradish peroxidase-conjugated sheep anti-FITC secondary antibodies (Bio-Rad, Muenchen, Germany) were diluted 1:5000 in PBS-T; the antibodies were incubated on the plate 1 h at room temperature. After incubation, the plate was washed 3 times with PBS-T. Subsequently, the substrate solution was prepared as follows: 10 mg of 3,3',5,5'-tetramethylbenzidine (TMB) (Roth, Karlsruhe, Germany) was dissolved in 1 ml dimethylsulfoxide (DMSO), 62 µL from TMB solution were mixed with 9 mL distilled water, 1 mL 1 M sodium acetate buffer (pH 6) and 10 µL 3 % hydrogen peroxide. 100 µL of the prepared substrate solution were transferred to the according wells. Finally, the reaction was stopped with 100 µL/well of 20 % H_2_SO_4_, and the plate was read at 450 nm (Multiskan GO, Thermo scientific, Germany).

**S4 Fig Materials and Methods: Treatment of inducible N2aTau^K18ΔK280^ cells with d-peptides**

Cell suspensions of inducible N2aTau^K18ΔK280^ cells were distributed on 6-well plates (4 mL per well) and incubated in the presence of 0.0005 % Thioflavin S, 1 µg/mL doxycyclin (except for the uninduced negative control) and 10 to 60 µM peptides (except for the untreated positive control) for 4 days at 37 °C. After incubation, the floating and adherent cells were combined, pelleted (5 min, 295 g), washed once with PBS and 10.000 events were counted in a BD FACSAria cytometer. Cells with high ThS-fluorescence indicating the presence of Tau-aggregates were measured in the FITC-channel (exc. 495 nm, em. 519 nm). The amount of ThS-positive cells in the induced but compound untreated sample was set to 100 %.

**S4 Supplementary Literature**

[1] Barghorn S, Biernat J and Mandelkow E. (2005) Purification of recombinant tau protein and preparation of Alzheimer-paired helical filaments in vitro. Methods Mol Biol. 299, 35-51.
